# Comparison of the Oncological Efficacy Between Intraoperative Radiotherapy With Whole-Breast Irradiation for Early Breast Cancer: A Meta-Analysis

**DOI:** 10.3389/fonc.2021.759903

**Published:** 2021-12-17

**Authors:** Lin He, Jiejing Zhou, Yuhong Qi, Dongjie He, Canliang Yuan, Hao Chang, Qiming Wang, Gaiyan Li, Qiuju Shao

**Affiliations:** ^1^ Department of Radiotherapy, Tangdu Hospital, Air Force Military Medical University, Xi’an, China; ^2^ Cancer Center, Faculty of Health Sciences, University of Macau, Macau, Macau SAR, China

**Keywords:** intraoperative radiotherapy, whole-breast irradiation, early breast cancer, oncological efficacy, meta-analysis

## Abstract

**Background:**

Intraoperative radiotherapy (IORT) and whole-breast irradiation (WBI) are both effective radiotherapeutic interventions for early breast cancer patients undergoing breast-conserving surgery; however, an issue on whether which one can entail the better prognosis is still controversial. Our study aimed to investigate the 5-year oncological efficacy of the IORT cohort and the WBI cohort, respectively, and compare the oncological efficacy between the cohorts.

**Materials and Methods:**

We conducted a computerized retrieval to identify English published articles between 2000 and 2021 in the PubMed, the Web of Science, the Cochrane Library, and APA PsycInfo databases. Screening, data extraction, and quality assessment were performed in duplicate.

**Results:**

A total of 38 studies were eligible, with 30,225 analyzed participants. A non-comparative binary meta-analysis was performed to calculate the weighted average 5-year local recurrence-free survival (LRFS), distant metastasis-free survival (DMFS), and overall survival (OS) in the two cohorts, respectively. The LRFS, DMFS, and OS (without restriction on the 5-year outcomes) between the two cohorts were further investigated by a comparative binary meta-analysis. The weighted average 5-year LRFS, DMFS, and OS in the IORT cohort were 96.3, 96.6, and 94.1%, respectively, and in the WBI cohort were 98.0, 94.9, and 94.9%, respectively. Our pooled results indicated that the LRFS in the IORT cohort was significantly lower than that in the WBI cohort (pooled odds ratio [OR] = 2.36; 95% confidential interval [CI], 1.66–3.36). Nevertheless, the comparisons of DMFS (pooled OR = 1.00; 95% CI, 0.76–1.31), and OS (pooled OR = 0.95; 95% CI, 0.79–1.14) between the IORT cohort with the WBI cohort were both not statistically significant.

**Conclusions:**

Despite the drastically high 5-year oncological efficacy in both cohorts, the LRFS in the IORT cohort is significantly poorer than that in the WBI cohort, and DMFS and OS do not differ between cohorts.

## Research in Context

### Evidence Before This Study

We searched PubMed, without language and date restrictions, on July 1, 2021, by using a retrieval strategy: (early breast cancer) AND (breast-conserving surgery) AND ((intraoperative radiotherapy) OR (whole-breast irradiation)) AND survival. The use of IORT and WBI was supported by a growing body of high-quality clinical studies. Since the advent of WBI was earlier than that of IORT, WBI was utilized more frequently than IORT for early breast cancer patients undergoing breast-conserving surgery. All studies showed similar disease-free survival (DFS) and overall survival (OS) outcomes between the IORT cohort and the WBI cohort but the results for local control, i.e., local recurrence-free survival (LRFS), were divergent. The current degree of uncertainty might be attributable to the heterogeneity of irradiation techniques used, the eligibility criteria of participants, and the overall reporting time of outcomes. To diminish this heterogeneity, a non-comparative binary meta-analysis was conducted to assess the 5-year oncological efficacy of a single dose of 21 Gy IORT delivered during surgery (i.e., excluded as a tumor bed boost before WBI), and a 45–50 Gy WBI with a 10–16 Gy boost, respectively. However, for including the largest number of eligible studies in a comparative binary meta-analysis that investigated the comparison of the oncological efficacy between the cohorts, we did not restrict the radiotherapeutic strategy and the overall reporting time in the inclusion criteria.

### Added Value of This Study

This meta-analysis supported the use of IORT and WBI in clinical settings. The substantial to considerable heterogeneity of all included studies for the non-comparative meta-analysis gave the opportunity to better understand the importance of patient characteristics (e.g., mean age, tumor biology, histology, and molecular subtype) on prognostic outcomes. Our present study implicated the optimal 5-year oncological efficacy in the IORT cohort and the WBI cohort, and showed a significant decrease in the LRFS in the IORT cohort compared with the WBI cohort, despite no significant difference of DMFS and OS between the cohorts. Additionally, we emphasized the significance of choosing the most appropriate participants for the IORT cohort in the discussion section, because the local control failure was a devastating situation for the patients.

### Implications of all the Available Evidence

Although early breast cancer patients undergoing breast-conserving surgery combined with IORT or WBI can achieve great oncological efficacy and similar DMFS and OS between the cohorts, criteria are awaited to establish the delivery strategies of techniques and the conditions of the patient for IORT. The established criteria need to involve some easily attained parameters before surgery (e.g., age, menopausal status, tumor size, hormone receptor status, molecular subtypes, and proliferation index), and the optimal dose and the delivery timeframe of IORT. The preoperative parameters should be stricter than the American Society of Radiation Oncology criteria for accelerated partial breast irradiation. About the irradiation strategy of IORT, two uncertainties require the answer. Is a single dose of 21 Gy the best dose of IORT? When the postoperative histopathology of the patient is at any of the following high risks, grade 3 tumor, lymph node-positive, and lymphatic vessel infiltration, whether IORT is considered as a tumor bed boost followed by WBI?

## Introduction

Female breast cancer has surpassed lung cancer to become the most common diagnostic cancer in 2020; among women, breast cancer ranks first for the incidence of new diagnostic cancer and cancer-related mortality worldwide, with an estimated 2.254 million new cases (24.5%) and 0.682 million new deaths (15.5%) (1). Breast cancer in different stages is tailored to use corresponding treatment strategies. Several randomized controlled trials (RCTs) (2–6) and a meta-analysis of the Early Breast Cancer Trialists’ Collaborative Group (EBCTCG) (7) revealed the equivalent overall survival (OS) of early breast cancer patients treated with breast-conserving surgery followed by whole-breast irradiation (WBI) and those undergoing mastectomy. Another meta-analysis of the EBCTCG found that WBI significantly reduced the long-term risk of any first recurrence (i.e., local–regional relapse or distant metastasis) and long-term mortality of early breast cancer patients (8). The above findings demonstrate that breast-conserving surgery followed by WBI, conventionally consisting of 45–50 Gy over 4.5–5 weeks with or without a tumor bed boost, has been widely accepted as the standard care for early breast cancer.

In the past two decades, with the deep understanding of tumor biology and the introduction of many modern high-precision technologies into radiotherapy, we have witnessed the rapid development of radiotherapy and realized several limitations of the current WBI (e.g., lengthy treatment schedule, radiation effect, and long-term toxicity), which severely affect the acceptability, accessibility, and practical management of WBI (9). Serious medical challenges occur every day in the transitioning countries with limited resources or in the areas with a substantial distance from a radiotherapy center within the transitioned countries. In this context, an accelerated partial breast irradiation (APBI) with an attenuated planning target volume and shortened treatment duration is introduced to solve these problems.

Intraoperative radiotherapy (IORT) is an innovative form of APBI and has been applied by several radiotherapeutic/oncological institutes to treat early breast cancer during the past decade. The European Institute of Oncology (Milan, Italy) uses IORT to surrogate the postoperative WBI; after completion of lumpectomy, an intraoperative single shot of radiation with at least of the same biologically equivalent dose is delivered to the breast volume where the tumor is previously located (10). By contrast, the TARGIT-IORT trial group utilizes IORT as a treatment alternative for WBI or the tumor bed boost; when postoperative histopathology finds any of the unsuspected high risks, i.e., grade 3 tumor, lymph node-positive, and lymphatic vessel infiltration, IORT is supplemented by WBI (11). IORT provides the following advantages: an excellent delineation of tumor bed under visual control, a good dose homogeneity, an excellent normal tissue sparing (12), improved quality of life of patients, avoidance of tumor growth between the duration from the completion of surgery to the beginning of adjuvant radiotherapy, and the averted radiation to heart and lungs when a shield between breast and the pectoralis muscle is positioned (13).

The standardized dose of IORT introduced by the European Institute of Oncology is 21 Gy (13). In fact, the intraoperative single dose of 21 Gy is biologically equivalent to 1.5–2.5 folds of the total dose of WBI with or without boost (14). Since the advent of WBI and IORT, increasing bodies of radiation/oncology centers have evaluated the oncological efficacy and the possible local side-effects of both techniques (9, 15–17). In the recent decade, many RCTs have centered on the comparison of local–regional control, distant metastasis, and OS between the early breast cancer patients undergoing WBI (defined as the WBI cohort) and those undergoing IORT (defined as the IORT cohort) (18–20). For example, end-of-study results of an RCT after a median follow-up of 12.4 years indicated that the IORT cohort had a 9% higher of ipsilateral breast tumor recurrence rate than the WBI cohort (hazard ratio [HR] = 4.62; 95% CI, 2.68–7.95; *P <*0.0001) (18). However, because of the diversity in demography, histopathology, and systematic treatment modality across different clinical studies, the reported results in the oncological efficacy of the cohorts, and the efficacy comparison between them are discordant. In this context, our present study aimed to settle this issue by performing a non-comparative binary meta-analysis to investigate the individually weighted average proportion of 5-year oncological efficacy in the WBI cohort and the IORT cohort, respectively, and a comparative binary meta-analysis to compare the oncological efficacy between the cohorts.

## Materials and Methods

This meta-analysis complied with the Preferred Reporting Items for Systematic Reviews and Meta-analyses (PRISMA) guidelines (21, 22). There was no need for Ethical or Institutional Review Board Approval for the study design due to the nature of our work.

### Literature Search

A computerized retrieval was conducted on July 9, 2021 in the PubMed, the Web of Science, the Cochrane Library, and the APA PsycInfo databases to identify English published articles. The following terms were used: ((“breast cancer[MesH]” AND early) OR (early breast tumor) OR (early breast tumour) OR (early breast carcinoma) OR (early breast cancer) OR (early-stage breast tumor) OR (early-stage breast tumour) OR (early-stage breast carcinoma) OR (early-stage breast cancer)) AND (((intra-operative OR intraoperative) AND (irradiation OR radiation OR radiotherapy)) OR (whole-breast irradiation) OR (whole breast radiotherapy)) AND (recurrence OR relapse OR reappearance OR metastasis OR survival).

### Inclusion and Exclusion Criteria

Clinical articles reporting the oncological efficacy of the WBI cohort or the IORT cohort, or comparing that between the cohorts was considered to be eligible. Additionally, potential studies with publication year from 2000 to 2021 were required to meet the following inclusion criteria: (1) populations—patients with early breast cancer; (2) treatment strategy—breast-conserving surgery plus WBI or IORT; (3) endpoints—local recurrence-free survival (LRFS), distant metastasis-free survival (DMFS), and/or OS. As a significantly decreased local recurrence (LR) rate in WBI with a boost compared with WBI without a boost (23), only the clinical studies that patients underwent 45–50 Gy WBI with a 10–16 Gy boost or a single dose of 21 Gy IORT were included into computing the weighted average proportion of 5-year oncological efficacy in the WBI cohort or the IORT cohort, respectively. Of note, comparison of the oncological efficacy between the cohorts did not merely focus on the 5-year outcomes and did not confine to the same delivery paradigm (i.e., the hypofractionated radiotherapy or WBI without a boost was also eligible to the WBI cohort, and IORT as a boost followed by WBI when postoperative histopathology experienced any of the previously mentioned high risks was also involved in the IORT cohort). Besides, retrieved citations that emerged any of the following criteria were removed: (1) article type—reviews, case reports, case series that involved less than 10 patients, editorials, letters, comments, and conference papers; (2) populations—female patients who underwent neoadjuvant treatment prior to breast-conserving surgery, and male patients; (3) the studies for calculating the weighted average proportion did not apply the above-mentioned delivery paradigm and/or did not report the 5-year outcomes; and (4) overlapping study populations.

### Data Extraction and Quality Assessment

We extracted the following data from the included studies by using a standardized form: (1) study characteristics—family name of the first author, publication year, recruitment duration, original nation, study type, number of patients, and median follow-up; (2) demographic characteristics—mean age, molecular subtype, tumor size, lymph node status, and tumor grade; and (3) outcome characteristics—the event number of LRFS, DMFS, and OS in WBI cohort and/or IORT cohort. The LRFS was defined as the time from diagnosis to the time of tumor reappearing at the site of the surgical resection. We defined the DMFS as the time from diagnosis to the time of any recurrence of carcinoma to distant organs and/or tissues. The OS was defined as the time from diagnosis to last follow-up or time of death. Quality assessment of the analyzed studies for the comparative meta-analysis was judged by drawing figures of the risk of bias summary and the risk of bias graph with Review Manager 5.4 (https://training.cochrane.org/online-learning/core-software-cochrane-reviews/revman). The included studies only for calculating the weighted average proportion did not require quality assessment due to the non-comparative data that were impossible or fair to assign equal weight to different quality aspects. Two co-authors (JZ and QW) independently assessed the literature search, study selection, and data extraction. If there were any inconsistencies, they were addressed by a third co-author (YQ).

### Data Synthesis and Statistical Analysis

The primary outcome for calculating the weighted average proportion of the 5-year oncological efficacy in the cohorts included the rates of LRFS, DMFS, and OS with corresponding 95% confidence intervals (CIs), and for comparing the oncological efficacy between the cohorts was presented as odds ratio (OR) with 95% CIs. The crude proportions or ORs were independently calculated and then pooled together. The number of events, if it was not provided in the article, was calculated in terms of the endpoint percentage or other relevant information. The heterogenicity that implicated the degree of variability in results across the included studies was assessed by Cochran’s Q test and Higgins I^2^ statistic test (24); *P <*0.10 suggested significant heterogeneity, and different cutoff intervals of I^2^ values at 0–25%, 25–50%, 50–75%, and 75–100% corresponded to nonsignificant, moderate, substantial, and considerable heterogeneity, respectively. When the heterogeneity test was not statistically significant (*P* ≥0.10), a binary fixed-effect model, the Mantel–Haenszel method was used to pool data, and if not so, a binary random-effect model, the Dersimonian–Laird method was employed (25). The publication bias in the comparative meta-analysis was evaluated by an Egger’s test with a significant level of *P <*0.05. All statistical analyses were performed by the software Open Meta-Analyst (http://www.cebm.brown.edu/openmeta/download.html).

## Results

### Literature Search

**Figure 1:**
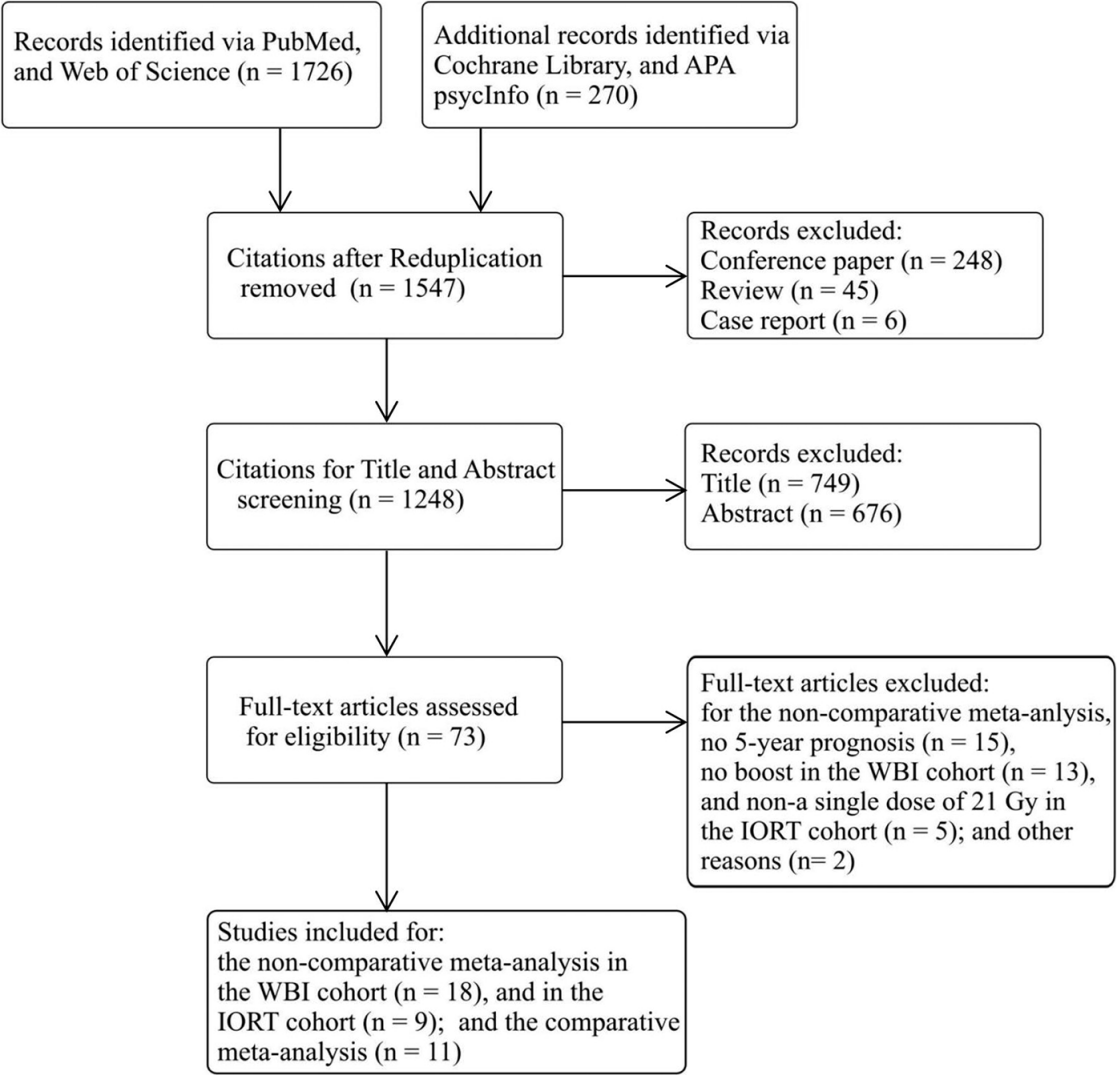
PRISMA flow diagram of study selection. IORT, intraoperative radiotherapy; WBI, whole-breast irradiation.

A PRISMA flow diagram of the literature screening selection is outlined in **Figure 1**. A total of 1,996 citations were obtained from the PubMed, the Web of Science, the Cochrane Library, and the APA PsycInfo databases, and 449 reduplications, 248 conference papers, 45 reviews, and 6 case reports were excluded. The remaining 1,248 citations were evaluated by title and abstract screening, and 1,422 of them were removed; fundamental characteristics of the abstracts were judged with respect to the inclusion and exclusion criteria, and 73 full-length articles were chosen. After full-text scrutinization, 35 of them were further omitted for the following reasons: (1) no provision of the 5-year outcomes in 15 potential articles for calculating the weighted average proportion; (2) no application of a 10–16 Gy boost to the patient in 13 potential studies for calculating the weighted average proportion in the WBI cohort; (4) no usage of a single dose of 21 Gy in five potential studies for calculating the weighted average proportion in the IORT cohort; and (5) 2 articles with other reasons. Ultimately, 38 articles with 30,225 early breast cancer patients were involved (9–12, 15–20, 26–53), in which 18 exclusively analyzed the 5-year oncological efficacy in the WBI cohort (15, 16, 26–41), 9 exclusively analyzed that in the IORT cohort (9, 12, 17, 42–47), and 11 compared the oncological efficacy between the cohorts (10, 11, 18–20, 48–53). Three of the 11 studies for the comparative meta-analysis had the available data to calculate the weighted average 5-year oncological efficacy in the non-comparative meta-analysis (10, 18, 20).

### Characteristics of the Studies Included for Meta-Analysis

The characteristics of the 38 included studies in the “study-level” analysis are presented in **Table 1**, and those in the “patient-level” analysis are summarized in **Table 2**. Nearly half of the studies (n = 18) were categorized as retrospective trials; the publication year ranged from 2001 to 2021 (median: 2016); the median value of the mean age calculated from all the available studies was 58.0 years (45.0–74.1), and that of the median follow-up was 5.8 years (2.5–12.4); Italy ranked first for the original nation of all the involved studies (n = 8). Thirty studies provided the information of breast cancer subtype, with the median proportion of Luminal breast cancer of 90.0% (39.4–98.3); 31 studies provided the information of tumor size, with the median proportion of T1 stage of 81.9% (50.4–95.2); 28 studies provided the information of lymph node status, with the median proportion of N0 stage of 81.0% (0.0–100.0); and 24 studies provided the information of tumor grade, and the median proportion of tumor of grade 1 was 26.9% (9.2–60.1). Additionally, **Table 1** provides the event number of LR, DM, and death from all the analyzed studies.

**Table 1:** Characteristics of included studies in the “study-level” analysis.

Study (Published year)	Recruitment duration	Original nation	Follow-up (y)	Age (y)	Total sample (N)		LR (n)		DM (n)		Death (n)		Ref.
WBI													
Strnad (2016)	2004–2009	Germany	10.2	62.0	551		5		5		25		(15)
Bosma (2021)	2004–2011	The Netherlands	10.0	NA	2421		26		/		/		(16)
Lee (2016)	2007–2010	South Korea	6.3	50.0	379		5		9		4		(26)
Meattini (2015)	2005–2013	Italy	4.8	74.1	58		2		1		4		(27)
Livi (2015)	2005–2013	Italy	5.3	NA	260		4		5		9		(28)
Meattini (2020)	2005–2013	Italy	10.7	NA	260		3		6		8		(29)
Lee (2016)	2007–2010	South Korea	6.8	50.0	330		/		10		/		(30)
Guinot (2007)	1996–2000	Spain	7.0	NA	125		5		/		9		(31)
Keller (2012)	2003–2010	USA	2.6	58.0	946		19		/		/		(32)
Frazier (2001)	1980–1989	USA	9.7	55.0	552		15		62		68		(33)
Yoshida-Ichikawa (2021)	2006–2010	Japan	9.4	NA	186		/		/		1		(34)
Ha (2013)	2001–2008	South Korea	7.3	46.0	214		2		/		1		(35)
Bartelink (2001)	1989–1996	The Netherlands	5.1	54.8	2661		114		346		239		(36)
Hirata (2017)	1993–2010	Japan	9.4	52.0	121		/		/		5		(37)
Poortmans (2008)	1989–1996	Belgium	10.8	54.8	2661		90		/		/		(38)
Zhao (2017)	2006–2007	China	10.2	45.3	54		2		/		2		(39)
Kim (2005)	1994–2001	South Korea	3.9	45.0	605		17		53		28		(40)
You (2020)	2004–2014	China	NA	NA	1124		28		/		34		(41)
IORT													
Baatjes (2012)	2002–2005	South Africa	7.0	55.0	39		2		/		4		(9)
Kawamura (2015)	2007–2010	Japan	6.0	65.0	32		0		0		1		(17)
Takanen (2017)	2006–2016	Italy	5.2	NA	772		35		21		45		(42)
Lemanski (2013)	2004–2007	France	6.0	72.0	42		4		/		/		(12)
Chowdhry (2018)	2011–2017	USA	2.5	67.0	109		4		/		8		(43)
Hanna (2014)	2004–	Brazil	4.2	58.3	152		6		/		3		(44)
Veronesi (2010)	2000–2008	Italy	3.0	58.0	1822		/		/		47		(45)
Valente (2021)	2007–2013	United Arab Emirates	5.1	68.0	477		31		2		/		(46)
Bonzano (2018)	2009–2011	Italy	7.6	67.0	134		5		/		6		(47)
IORT *vs.* WBI					IORT	WBI	IORT	WBI	IORT	WBI	IORT	WBI	
Gunay (2019)	2013–2017	Turkey	3.0	51.0	98	99	0	2	/	/	/	/	(48)
Guenzi (2018)	2009–2012	USA	6.0	NA	235	235	8	1	/	/	23	12	(49)
Vaidya (2020)	2004–2019	UK	NA	NA	581	572	41	19	26	18	19	13	(50)
Zhou (2012)	2007–2011	China	2.7	NA	72	71	2	1	2	2	1	0	(19)
Orecchia (2021)^*^	2000–2007	Italy	12.4	NA	651	654	/	/	46	54	98	95	(18)
Lei (2020)	1998–2013	China	NA	NA	686	2744	/	/	/	/	13	72	(51)
Oliver Guillén (2021)	2012–2017	Spain	3.1	66.1	217	208	2	1	2	1	3	6	(52)
Veronesi (2013)^*^	2000–2007	Italy	5.8	NA	651	654	21	4	33	35	34	31	(10)
Vaidya (2020)	2000–2012	UK	8.6	NA	1140	1158	24	11	/	/	42	68	(11)
Abo-Madyan (2019)^*^	2002–2012	Germany	8.5	66.2	90	90	0	1	3	2	5	6	(20)
Vaidya (2010)	2000–2010	UK	4.5	63.0	1113	1119	6	5	/	/	/	/	(53)

^*^These three studies also provide available data for analyzing the 5-year prognosis of patients treated with whole-breast irradiation or intraoperative radiotherapy.

DM, distant metastasis; IORT, intraoperative radiotherapy; LR, local recurrence; NA, not assessed; WBI, whole-breast irradiation; y, year.

**Table 2:** Characteristics of included studies in the “patient-level” analysis.

Characteristic	Studies, No. (%) (N = 38)	Analyzed participants, No. (%) (N = 30,225)
Study type		
Retrospective trial	18 (47.4)	12,637 (41.8)
Prospective trial	7 (18.4)	725 (2.4)
Randomized controlled trial	11 (28.9)	14,403 (47.7)
Case-control study	1 (2.6)	2421 (8.0)
Case series study	1 (2.6)	39 (0.1)
Publication year, median (range), y	2,016 (2001–2021)	
Mean age, median (range), y^*^	58.0 (45.0–74.1)	
Median follow-up, median (range), y^*^	5.8 (2.5–12.4)	
Meta-analysis^†^		
Exclusive IORT analysis	9 (23.7)	3579 (11.8)
Exclusive WBI analysis	18 (47.4)	13,508 (44.7)
IORT *vs.* WBI	11 (28.9)	13,138 (43.5)
Original nation		
USA	4 (10.5)	2,077 (6.9)
UK	3 (7.9)	5,683 (18.8)
Italy	8 (21.1)	5,916 (19.6)
China	4 (10.5)	4,751 (15.7)
Japan	3 (7.9)	339 (1.1)
Germany	2 (5.3)	731 (2.4)
South Korea	4 (10.5)	1,528 (5.1)
South Africa	1 (2.6)	39 (0.1)
France	1 (2.6)	42 (0.1)
Spain	2 (5.3)	550 (1.8)
The Netherlands	2 (5.3)	5,082 (16.8)
United Arab Emirates	1 (2.6)	477 (1.6)
Belgium	1 (2.6)	2,661 (8.8)
Turkey	1 (2.6)	197 (0.7)
Brazil	1 (2.6)	152 (0.5)
Classification of Breast cancer subtype		
Yes	30 (78.9)	22,794 (75.4)
No	8 (21.1)	7,431 (24.6)
Proportion of Luminal BC, median (range), %^*^	90.0 (39.4–98.3)	
Classification of T stage		
Yes	31 (81.6)	26,515 (87.7)
No	7 (18.4)	3,710 (12.3)
Proportion of T1 stage, median (range), %^*^	81.9 (50.4–95.2)	
Classification of N stage		
Yes	28 (73.7)	21827 (72.2)
No	10 (26.3)	8398 (27.8)
Proportion of N0 stage, median (range), %^*^	81.0 (0.0–100.0)	
Classification of Tumor Grade		
Yes	24 (63.2)	18,964 (62.7)
No	14 (36.8)	11,261 (37.3)
Proportion of G1, median (range), %^*^	26.9 (9.2–60.1)	

^*^The calculation of median value is based on the provided data from included studies.

^†^Meta-analysis includes the estimated proportions of 5-year prognosis of patients treated with whole-breast irradiation or intraoperative radiotherapy and the pooled results of comparing the prognosis between patients treated with whole-breast irradiation group and those treated with intraoperative radiotherapy.

WBI, whole-breast irradiation; IORT, intraoperative radiotherapy; Luminal BC, Luminal breast cancer.

### LRFS

**Figure 2:**
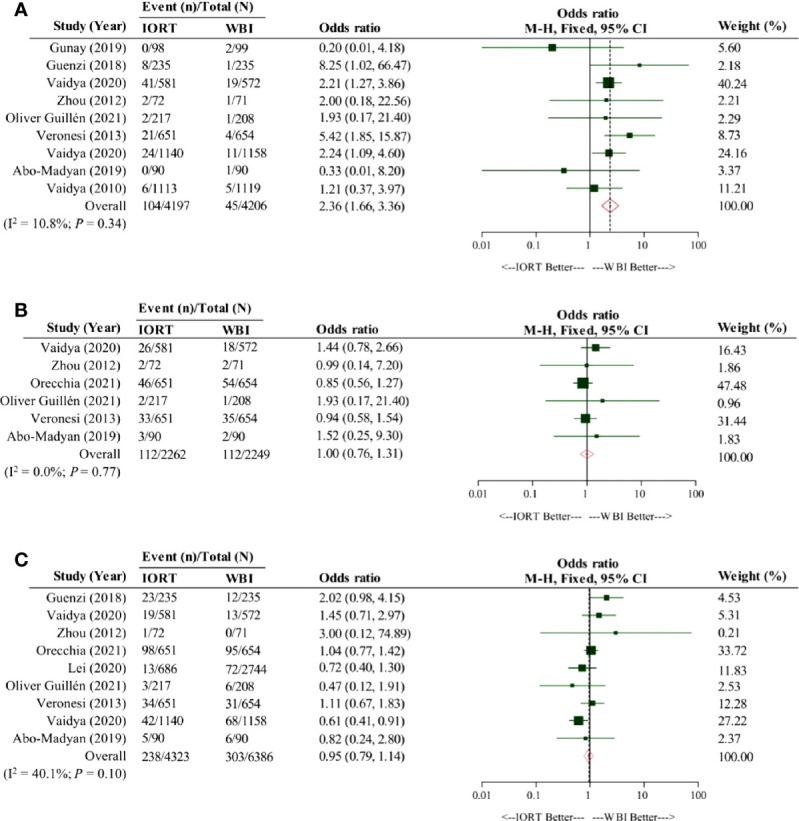
Pooled forest plot for comparison of the oncological efficacy between intraoperative radiotherapy cohort and whole-breast irradiation cohort. Panel **(A)** shows the pooled forest plot for comparison of local relapse-free survival, Panel **(B)** shows the pooled forest plot for comparison of distant metastasis-free survival, and Panel **(C)** shows the pooled forest plot for comparison of overall survival. IORT, intraoperative radiotherapy cohort; WBI, whole-breast irradiation cohort.

We collected 10 (9, 10, 12, 17, 20, 42–44, 46, 47) and 17 (10, 15, 16, 20, 26–29, 31–33, 35, 36, 38–41) articles for the calculation of the weighted average 5-year LRFS rates in the IORT cohort and the WBI cohort, respectively; the pooled result indicated them were 96.3% (95% CI, 94.9–97.7%) and 98.0% (95% CI, 97.3–98.6%), respectively (**Table 3**). There were nine studies included for the comparison of LRFS rate between the cohorts (10, 11, 19, 20, 48–50, 52, 53). The pooled result showed a significantly lower LRFS rate in the IORT cohort than the WBI cohort (OR = 2.36; 95% CI, 1.66–3.36) (**Figure 2A**).

**Table 3:** The weighted average proportion of 5-year oncological efficacy *via* meta-analysis with random-effect model.

Analysis	Estimated proportion (95% CI) (%)	Included studies (N)	Event/Total (N)	Heterogeneity test	
				I^2^	*P*-value
IORT					
5-year LRFS	96.3 (94.9–97.7)	10	2,393/2,501	66.0%	0.002
5-year DMFS	96.6 (94.3–98.9)	6	2,574/2,679	92.4%	<0.001
5-year OS	94.1 (91.8–96.5)	10	4,207/4,458	90.0%	<0.001
WBI					
5-year LRFS	98.0 (97.3–98.6)	17	13,271/13,615	84.8%	<0.001
5-year DMFS	94.9 (92.2–97.6)	12	6,466/7,054	96.7%	<0.001
5-year OS	94.9 (93.1–96.7)	17	7,979/8,548	94.8%	<0.001

IORT, intraoperative radiotherapy; WBI, whole-breast irradiation; LR, local recurrence; DM, distant metastasis.

### DMFS

Six (10, 17, 18, 20, 42, 46) and 12 (10, 15, 18, 20, 26–30, 33, 36, 40) studies were respectively involved for the analysis of the weighted average 5-year DMFS rates in the IORT cohort and the WBI cohort. Our pooled result showed that the weighted average 5-year DMFS rate in the IORT cohort (96.6% [95% CI, 94.3–98.9%]) outnumbered that in the WBI cohort (94.9% [95% CI, 92.2–97.6%]) (**Table 3**). Based on the original data from six analyzed articles (10, 18–20, 50, 52), the pooled result suggested that the DMFS rate in the IORT cohort was not significantly different from that in the WBI cohort (OR = 1.00; 95% CI, 0.76–1.31) (**Figure 2B**).

### OS

The OS of early breast cancer patients undergoing IORT or WBI was the greatest concerning issue for which ten (9, 10, 17, 18, 20, 42–45, 47) and 17 (10, 15, 18, 20, 26–29, 31, 33–37, 39–41) studies were respectively obtained to calculate the weighted average 5-year OS rates in the IORT cohort and the WBI cohort; the pooled result revealed that the weighted average 5-year OS rate in the IORT cohort (94.1% [95% CI, 91.8–96.5%]) was nearly equivalent to that in the WBI cohort (94.9% [95% CI, 93.1–96.7%]) (**Table 3**). By analyzing the crude data from nine included studies (10, 11, 18, 19, 49–53), our pooled result consistently implicated a similar OS between the cohorts (OR = 0.95; 95% CI, 0.79–1.14) (**Figure 2C**).

### Risk of Bias

**Figure 3:**
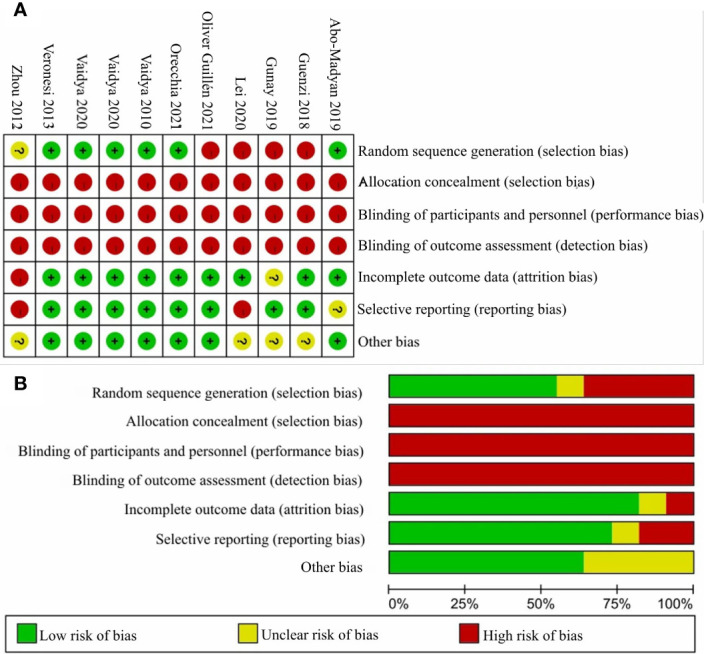
The judgments of risk of bias summary and risk of bias graph. Panel **(A)** shows the judgement of risk of bias summary and Panel **(B)** shows the judgement of risk of bias graph.

The 11 studies for the comparative meta-analysis were combined to judge each risk of bias domain. The risk of bias summary and the risk of bias graph are shown in **Figures 3A, B**. We moreover assessed the detailed risk of bias in the 11 articles (**Supplementary Materials: eTable 1**, page 1–2). Overall, all clinical trials were at high risk of bias concerning the allocation concealment, the blinding of participants and personnel, and also the blinding of outcome assessment.

### Publication Bias

The publication bias results in the comparison of LRFS, DMFS, and OS between the cohorts in terms of the Egger’s test all were statistically nonsignificant (*P* = 0.474, 0.269, and 0.680, respectively) (**Supplementary Materials: eTable 2**, page 2). The results indicated that no publication bias existed in all comparative meta-analysis.

## Discussion

The non-comparative meta-analysis demonstrates the outstanding 5-year oncological efficacy in early breast cancer patients who undergo breast-conserving surgery combined with a single 21 Gy IORT or a 45–50 Gy WBI plus a 10–16 Gy boost. The 5-year weighted average LRFS, DMFS, and OS in the IORT cohort were 96.3, 96.6, and 94.1%, respectively, and in the WBI cohort were 98.0, 94.9, and 94.9%, respectively. As suggested by Vaidya and colleagues, the rates of overall complications (17.6% in the IORT cohort vs 15.5% in the WBI cohort; *P* = 0.19) and major toxicity (3.3% in the IORT cohort *vs* 3.9% in the WBI cohort; *P* = 0.44) were similar in two cohorts (53). Nonetheless, some women after WBI always suffer from breast atrophy, malformation of the irradiated breast, pigmentation, and rough skin (19). By contrast, the quality of life (e.g., cosmesis, and breast pain) of patients and the economization of healthcare system resources are optimized by the introduction of IORT (54, 55). An estimated savings of around £15 million is ascertained annually with the use of IORT in countries like the UK (56). The popularity of IORT for the treatment of early breast cancer is witnessed in the USA, with a tenfold increment from 2010 to 2013 in light of an analysis in the Surveillance, Epidemiology, and End Results Program database (51). Thanks to its unique advantages, IORT is more desirable than WBI in early breast cancer patients, even in the face of a hypothetically poorer local control.

LRFS has received great attention when planning the treatment strategy for early breast cancer patients. Our findings confirm the inferiority of LRFS in the IORT cohort compared with the WBI cohort, which is consistent with the conclusion of a retrospective observational study with a median follow-up of six years (49) but is marginally discordant to that of the phase 3 TARGIT-A RCT (50) that only shows a statistically significant trend. Demographically, the proportional distributions of Luminal breast cancer, T1 grade, N0 grade, and HER2-negative status in the cohorts of the two studies were well-balanced. In contrast, tumors of grade 2 predominantly accounted for 3/4 cases of the cohorts in the observational study (74.9% in the IORT cohort and 75.7% in the WBI cohort), but tumors of grade 1 were the main part of both cohorts in the TARGIT-A RCT (56.5% in the IORT cohort and 63.8% in the WBI cohort); WBI used in the observational study, in fact, was a hypofractionated schedule, i.e., hypofractionated radiotherapy, and the TARGIT-A RCT applied IORT as an intraoperative boost to the patients when they had any of high risks. As such, the difference in tumor grade and irradiation delivery may influence the incidence of LRFS. Given no heterogeneity among all studies (I^2^ = 10.8%; *P* = 0.34) (**Figure 2A**), the present study concludes that in the significantly poorer LRFS of early breast cancer patients undergoing IORT than WBI.

Clear evidence for no impact of the inferior LRFS in the IORT cohort on the DMFS, cancer-specific survival, and OS exists (10, 18, 51, 57). Our present results mirror these data, that is, an insignificant difference of DMFS and OS between the cohorts. The weighted average 5-year OS in both cohorts was nearly equivalent (94.1% in the IORT cohort and 94.9% in the WBI cohort, respectively); however, the weighted average 5-year DMFS in the IORT cohort (96.6%) was numerically greater than that in the WBI cohort (94.3%), corresponding to an absolute excess of 2.3% weighted average 5-year DMFS in the IORT cohort. The inconsistent DMFS outcomes between the non-comparative meta-analysis and the comparative meta-analysis may have the following explanations: (1) imbalanced tumor histological distribution, and (2) diverse radiotherapy doses and irradiation delivery techniques across all included studies for the two meta-analyses. To obviate the interference of different irradiation strategies, we conducted a subgroup analysis that compared the difference of DMFS between the IORT cohort and the WBI cohort, with the delivery strategy mapping to the non-comparative meta-analysis, and still observed that the difference was not statistically significant (OR = 0.90; 95%CI, 0.66–1.22) (**Supplementary Materials: eFigure 1**, page 2).

Albeit a decreased LRFS in the IORT cohort cannot compromise the DMFS and OS of patients, the improper treatment selection-caused local control failure in itself is an undesirable situation for patients and an additional economic burden to the healthcare system resources. It is therefore needed to determine which patients undergoing IORT are at high risk or at very low risk to develop LR. For the patients at high risk of LR, WBI with a boost or IORT supplemented by WBI is the more suitable alternative. Veronesi et al. (10) assessed the association between the characteristics of the patient in the IORT cohort and LR and identified that tumor size >2 cm, tumor of grade 3, positive lymph nodes ≥4, and triple-negative breast cancer were the high-risk factors for LR. An unplanned analysis in the long-term ELIOT phase 3 equivalence RCT (18) proposed a criterion for the very low-risk group: well-differentiated Luminal A tumor with a proliferation index (Ki-67) <14%, and tumor size <1 cm; the 5-year, 10-year, and 15-year rates of ipsilateral breast tumor recurrence in the IORT cohort and the WBI cohort were all very low and not significantly different (HR = 1.97; 95% CI, 0.36–10.8; *P* = 0.45). However, the definitive criteria for the high-risk group and the very low-risk group need to be verified in several independent datasets before they can be considered as any reliable statement in the clinical guidelines and consensus.

There were some limitations in the present study. First, the majority of analyzed studies for the non-comparative meta-analysis were performed retrospectively, which might indicate other biases due to the data collection and subject selection. Second, because the characteristics of the patient across the included studies for the non-comparative meta-analysis were divergent, the heterogeneity test indicated substantial to considerable heterogeneity, and thus the random-effect model was used to pool the data. Furthermore, we did not perform the subgroup analysis in terms of the high-risk group or the very low-risk group due to the limited data. Lastly, our study was absent from the analysis of long-term toxicity between the cohorts.

### Conclusions

This study demonstrates the optimal 5-year LRFS, DMFS, and OS in early breast cancer patients who undergo IORT or WBI. Additionally, the LRFS in the IORT cohort is significantly lower than that in the WBI cohort, while the DMFS and OS between the cohorts are devoid of significant difference.

## Data Availability Statement

The original contributions presented in the study are included in the article/**Supplementary Material**. Further inquiries can be directed to the corresponding author.

## Author Contributions

LH: Writing manuscript, Statistical analysis. JZ: Writing manuscript, data collection. YQ: Writing manuscript, supervision. DH: Table drawing. CY: Figure drawing. HC: Validity. QW: Supervision. GL: Data collection. QS: Conception/Design, final approval of manuscript. All authors contributed to the article and approved the submitted version.

## Conflict of Interest

The authors declare that the research was conducted in the absence of any commercial or financial relationships that could be construed as a potential conflict of interest.

## Publisher’s Note

All claims expressed in this article are solely those of the authors and do not necessarily represent those of their affiliated organizations, or those of the publisher, the editors and the reviewers. Any product that may be evaluated in this article, or claim that may be made by its manufacturer, is not guaranteed or endorsed by the publisher.

## Abbreviations

RCTs, randomized controlled trials; EBCTCG, Early Breast Cancer Trialists’ Collaborative Group; OS, overall survival; WBI, whole-breast irradiation; APBI, accelerated partial breast irradiation; IORT, intraoperative radiotherapy; PRISMAl, Preferred Reporting Items for Systematic Reviews and Meta-analyses; LRFS, local recurrence-free survival; DMFS, distant metastasis-free survival; LR, local recurrence; CIs, confidence intervals; OR, odds ratio.
