# Supplementary material for: Comparison of the Oncological Efficacy Between Intraoperative Radiotherapy With Whole-Breast Irradiation for Early Breast Cancer: A Meta-Analysis
Source: Front Oncol. 2021 Dec 17;11:759903. doi: 10.3389/fonc.2021.759903 (PMC8718609; doi:10.3389/fonc.2021.759903)
Supplement: Supplementary file 1 [file DataSheet_1.docx]

| **Risk of bias** | **Risk of bias summary** | **Proportion of low risk (%)** |
| --- | --- | --- |
| **Random sequence generation** | Six studies are described as randomized. One study does not provide sufficient information to accurately assess the method of random sequence generation, therefore is at unclear risk of bias. The remained four studies are at high risk of bias. | 50-75 |
| **Allocation concealment** | All studies do not provide details of allocation concealment, and therefore, they are at high risk of bias. | 0 |
| **Blinding of participants and personnel** | All studies do not provide details of blinding of participants and personnel, and therefore, they are at high risk of bias. | 0 |
| **Blinding of outcome assessment** | All studies do not provide details of blinding of outcome assessments, and therefore, they are at high risk of bias. | 0 |
| **Incomplete outcome data** | Nine studies are generally free of attrition bias. One study does not provide sufficient information to accurately assess the method of incomplete outcome data, therefore is at unclear risk of bias. The remained one studies are at high risk of bias. | 75-100 |
| **Selective reporting** | Eight studies are generally free of reporting bias. One study does not provide sufficient information to accurately assess the method of selective reporting, therefore is at unclear risk of bias. The remained two studies are at high risk of bias. | 50-75 |
| **Other bias** | Seven studies are free of other bias, but the other four studies are at unclear risk of bias. | 50-75 |

**eTable 1. The detailed risk of bias assessments.**

| **Analyzed label** | ***P* value^*^** |
| --- | --- |
| LRFS | 0.474 |
| DMFS | 0.269 |
| OS | 0.680 |
| Subgroup analysis for DMFS | 0.381 |

**eTable 2. The publication bias by Egger’s test.**

*Significant level: *P* < 0.05.

Abbreviations: LRFS, local recurrence-free survival; DMFS, distant metastasis-free survival; OS, overall, survival.


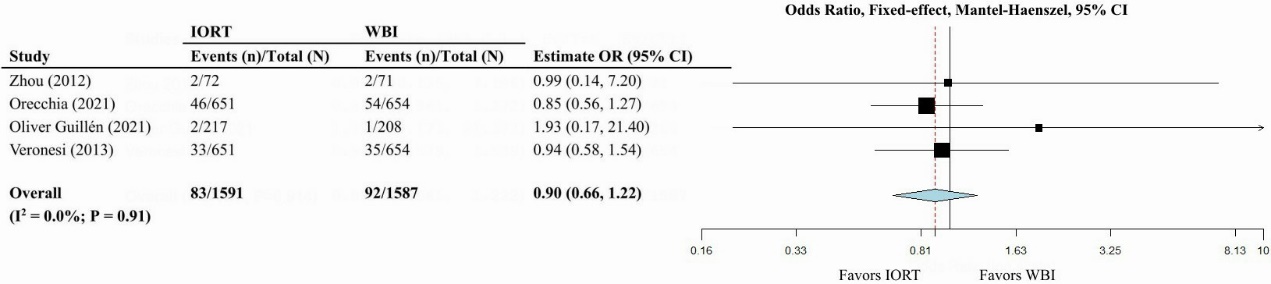


**eFigure 1. Pooled forest plot for comparison of distant metastasis-free survival between intraoperative radiotherapy cohort with 21 Gy and whole-breast irradiation cohort with 46-50 Gy plus a 10-16 Gy boost.**

Abbreviations: IORT, intraoperative radiotherapy cohort; WBI, whole-breast irradiation cohort.
